# Guidance for the Interpretation of Long-Acting Cabotegravir and Rilpivirine Concentrations Based on Real-World Therapeutic Drug Monitoring Data and Documented Failures

**DOI:** 10.1093/ofid/ofae023

**Published:** 2024-01-16

**Authors:** Paul Thoueille, Matthias Cavassini, Monia Guidi, Thierry Buclin, François R Girardin, Laurent A Decosterd, Catia Marzolini

**Affiliations:** Service and Laboratory of Clinical Pharmacology, Department of Laboratory Medicine and Pathology, Lausanne University Hospital and University of Lausanne, Lausanne, Switzerland; Service of Infectious Diseases, Department of Medicine, Lausanne University Hospital and University of Lausanne, Lausanne, Switzerland; Service and Laboratory of Clinical Pharmacology, Department of Laboratory Medicine and Pathology, Lausanne University Hospital and University of Lausanne, Lausanne, Switzerland; Centre for Research and Innovation in Clinical Pharmaceutical Sciences, Lausanne University Hospital and University of Lausanne, Lausanne, Switzerland; Institute of Pharmaceutical Sciences of Western Switzerland, University of Geneva, University of Lausanne, Geneva, Switzerland; Service and Laboratory of Clinical Pharmacology, Department of Laboratory Medicine and Pathology, Lausanne University Hospital and University of Lausanne, Lausanne, Switzerland; Service and Laboratory of Clinical Pharmacology, Department of Laboratory Medicine and Pathology, Lausanne University Hospital and University of Lausanne, Lausanne, Switzerland; Service and Laboratory of Clinical Pharmacology, Department of Laboratory Medicine and Pathology, Lausanne University Hospital and University of Lausanne, Lausanne, Switzerland; Service and Laboratory of Clinical Pharmacology, Department of Laboratory Medicine and Pathology, Lausanne University Hospital and University of Lausanne, Lausanne, Switzerland; Division of Infectious Diseases and Hospital Epidemiology, University Hospital Basel, University of Basel, Basel, Switzerland; Department of Molecular and Clinical Pharmacology, Institute of Translational Medicine, University of Liverpool, Liverpool, United Kingdom

**Keywords:** cabotegravir, guidance, long-acting, rilpivirine, TDM

## Abstract

The interpretation of long-acting cabotegravir and rilpivirine concentrations is complicated by the lack of consensus on the threshold to consider. Building on real-world therapeutic drug monitoring data and documented virologic failures, this article provides a reappraisal of the existing thresholds and guidance for the interpretation of cabotegravir and rilpivirine concentrations.

## Teaser

There is no established threshold for the interpretation of long-acting cabotegravir/rilpivirine concentrations. Building on real-world TDM data and documented virologic failures, this article provides a reappraisal of the existing thresholds, and guidance for the interpretation of cabotegravir and rilpivirine concentrations.

Long-acting (LA) injectable cabotegravir and rilpivirine are now approved in several countries as a complete regimen for the treatment of human immunodeficiency virus (HIV) infection. This new treatment paradigm offers the perspective to improve the quality of life of people with HIV (PWH) by reducing the stigma, the fear of disclosure, and the anxiety about treatment adherence, while eliminating the reminder of HIV status inherent to daily oral drug intake. Although adherence to treatment may become less problematic with injectables, emerging challenges may prevent the advantages of this treatment approach from being fully exploited. In particular, it was suggested that lower cabotegravir and rilpivirine concentrations could be associated with an increased risk of virologic failure (VF) [1]. This was specifically shown for low rilpivirine and/or cabotegravir concentrations at the end of the dosing interval (C_trough_) 4 weeks after the initial loading dose, in the presence of rilpivirine resistance mutations at baseline, HIV-1 subtype A6/A1, or body mass index (BMI) ≥30 (calculated as weight in kilograms divided by height in meters squared) in multivariable analyses from phase III data [1].

Even though the achievement of C_trough_ could be critical for treatment response, there is no consensus on the minimal efficacy threshold. Several thresholds have been used in clinical trials or recommended by experts. These include the protein-adjusted concentrations for 90% inhibition of viral replication (PAIC_90_), namely 166 and 12 ng/mL for cabotegravir and rilpivirine, respectively [2], or the corresponding 4 × PAIC_90_ thresholds, 664 ng/mL [3–5] and 50 ng/mL [6]. In addition, the HIV French resistance society recommends the upper limit of the first C_trough_ quartile (Q1_Ctrough_) derived from phase III trials, namely 1120 and 32 ng/mL for cabotegravir and rilpivirine, respectively [4, 7].

PWH on LA cabotegravir-rilpivirine are mostly monitored with viral load measurements, and in few settings also by therapeutic drug monitoring (TDM). However, the interpretation of LA cabotegravir-rilpivirine concentrations remains unclear owing to the lack of consensus on which threshold to refer to. Building on real-world TDM data from the Swiss HIV Cohort Study (SHCS) and reported VF in PWH with documented concentrations, we provide a reappraisal of the existing thresholds and a pragmatic guidance for the interpretation of cabotegravir-rilpivirine TDM data.

## METHODS

We analyzed LA cabotegravir-rilpivirine concentrations obtained from our Swiss nationwide observational study conducted between March 2022 and September 2023. Details of the study have been published elsewhere [8]. Briefly, PWH started on LA cabotegravir-rilpivirine after an oral lead-in underwent measurement of C_trough_ before each new injection. Demographic, clinical, and virologic data were documented at each visit.

Participants in the SHCS give general consent at inclusion in the cohort for the use of their clinical data for research purposes. Some PWH followed up in Lausanne and Geneva, Switzerland, were enrolled in a detailed pharmacokinetic substudy whose design has been approved by local ethical committees (project ID 2022–00619; approved by the Canton’s Ethics Committee, Lausanne).

We also compiled PWH with VF and documented drug measurements identified in our observational TDM study and in other cohorts based on a literature search. Specifically, we searched PubMed for publications and reviewed posters, abstracts, and reports presented at international conferences. We used the names of the compound or the search terms “long-acting antiretroviral” in combination with specific terms such as “virologic failure,” “drug concentration,” “TDM,” or “drug level.” References were selected if they reported cases of VF with documented cabotegravir and/or rilpivirine concentrations.

## RESULTS

In total, 1311 cabotegravir and rilpivirine TDM levels (214 during the initial oral lead-in period and 1097 after intramuscular injection) from 282 PWH (264 PWH under LA treatment and 18 under oral treatment at the end of the project) were collected. Specifically, the 1097 cabotegravir and rilpivirine levels from the 264 PWH were used to analyze the real-world distributions of LA cabotegravir-rilpivirine C_trough_ measurements after the initial loading injection (week 8) and over time (Supplementary Figure 1). The median follow-up duration was 34 weeks (range, 3–205 weeks), with a median of 4 samples (range, 1–17) collected per individual. Details on the proportion of PWH with cabotegravir and/or rilpivirine concentrations below the various thresholds are presented in Supplementary Table 1.

During the maintenance period, more than 40% of cabotegravir and almost 20% of rilpivirine C_trough_ measurements were below the thresholds of 1120 and 32 ng/mL (ie, Q1_Ctrough_), respectively. In addition, almost 50% of rilpivirine C_trough_ measurements were below the 50 ng/mL threshold, while approximately 15% of cabotegravir C_trough_ measurements were lower than 664 ng/mL (ie, 4 × PAIC_90_). Concentrations below the PAIC_90_ (ie, 166 and 12 ng/mL for cabotegravir and rilpivirine, respectively) were rare and represented approximately 1% of samples. Importantly, 11.4% and 43.9% of PWH had consecutive concentrations of LA cabotegravir-rilpivirine below the 4 × PAIC_90_ targets (ie, 664 and 50 ng/mL, respectively).

During our observational monitoring, 236 PWH (89.4%) remained fully virologically suppressed (viral load <50 copies/mL), 21 PWH (7.9%) had viral blips (isolated detectable viral load <200 copies/mL), and 5 PWH had VF (1.9%) (viral load ≥200 copies/mL) under LA cabotegravir-rilpivirine treatment. Table 1 summarizes the cases with VF and documented LA cabotegravir-rilpivirine levels identified in our observational study and in other cohorts [4, 9–11, 13]. It should be noted that VF was observed in one person with HIV from the SHCS whose drug levels were not measured until 6 months after discontinuation of treatment (not reported in Table 1).

**Table 1.** Characteristics of Study Participants With Reported Virologic Failure During Long-Acting Cabotegravir-Rilpivirine With Documented Drug Measurements at the Time of Failure^a^

Study Participant	Age, y	Sex/Gender	BMI^b^	HIV Subtype	Rebound, mo	Viral Load, Copies/mL	NNRTI Resistance	INSTI Resistance	Cabotegravir Level, ng/mL	Rilpivirine Level, ng/mL	Country [Reference]
1	38	Trans woman	29	B	9	15 000	101E, 138K	138K	2500	5	Netherlands [9]
2	49	Male	28	B	3	830 000	101E, 103R, 179D, 181 C	None	420	33	Netherlands [9]
3	50	Male	31.5	B	8	9400	101E	None	890	38	Netherlands [9]
4	56	Male	27	B	4	260/260 000^c^	101E, 138 K, 230L	138K	260	20	Netherlands [9]
5	43	Female	45.5	B	13	630	106A, 138K	138K	1400	20	Netherlands [9]
6	NA	Male	25.3	B	1	113 046	138A	138A	≍330	≍80	Spain [10]
7	NA	NA	29.4	NA	1	2820	None	None	701	28	France [11]
8	55	Male	23	CRF02	1	1500/5469	101E, 138 K, 188L,	97A, 138 K, 140S, 148H	2403	44	France [12]
9	NA	NA	NA	A6	NA	9000	138K	138 K, 140A, 148R	1910	91	Germany [13]
10	NA	NA	NA	B	NA	360	None	None	2240	48	Germany [13]
11	48	Female	31.1	02_AG	3	62 900	None	None	239^d^	4^d^	Switzerland [8]
12	47	Male	24.1	C	9	220	None	None	3069^e^	266^e^	Switzerland [8]
13	49	Male	23.9	C	3	17 000	None	None	1151^f^	52^f^	Switzerland [8]
14	33	Male	23.9	B	13	549	None	None	704^g^	55^g^	Switzerland [8]
15	NA	Male	<30	A6	27	887/1112	106A, 108I, 138G, 230L	138G, 155H	1730	79.5	Several countries [4]

Abbreviations: BMI, body mass index; HIV, human immunodeficiency virus; INSTI, integrase strand transfer inhibitor; NA, not available; NNRTI, nonnucleoside reverse-transcriptase inhibitor.

^a^All patients were virologically suppressed before initiation of cabotegravir-rilpivirine.

^b^BMI calculated as weight in kilograms divided by height in meters squared.

^c^Multiple entries indicate multiple measured viral load.

^d^Treatment was discontinued and reinitiated 4 months later using a longer needle. Subsequent cabotegravir levels were 272 and 402 ng/mL, and subsequent rilpivirine levels, 123 and 19 ng/mL.

^e^Virologic failure despite consistently therapeutic levels for both cabotegravir and rilpivirine.

^f^Subsequent measurements showed an unexpected 10-fold decrease in cabotegravir concentrations only 4 weeks after treatment discontinuation.

^g^The cabotegravir level at 9 months was 109 ng/mL.

Supplementary Figure 2 illustrates the cabotegravir and rilpivirine C_trough_ at the different time points and highlights the drug levels measured in SHCS participants with VF. Considering all VFs (in SHCS and other cohorts), those with concentrations below the 4 × PAIC_90_ thresholds were observed in 10 PWH (67%), while only 1 additional VF was observed when considering the cabotegravir Q1_Ctrough_ threshold. On the other hand, 4 PWH (27%) had VF even with concentrations above the highest thresholds; of those, 2 (50%) had the HIV subtype risk factor.

## DISCUSSION

Some SHCS patients with VF had low levels of cabotegravir and/or rilpivirine, while some had VF despite adequate levels, similar to what has been observed in other cohorts. However, when considering the distribution of cabotegravir C_trough_ measurements in our real-world study and cabotegravir concentrations in PWH with documented VF, it appears that the Q1_Ctrough_ threshold (ie, 1120 ng/mL) is too alarmist and may promote healthcare providers to unnecessarily discontinue LA treatment. Note that a large proportion of PWH below this threshold was also observed in phase III trials [4, 7]. In this context, the 4 × PAIC_90_ (ie, 664 ng/mL) target for cabotegravir should be retained, as supported also by Supplementary Figure 2, showing that the cabotegravir C_trough_ associated with VF tend to be <664 ng/mL rather than <1120 ng/mL. When considering the rilpivirine C_trough_ distribution, it appears that the Q1_Ctrough_ (ie, 32 ng/mL) is more appropriate than the 4 × PAIC_90_ (ie, 50 ng/mL). Nevertheless, VF is observed with rilpivirine concentrations below 50 ng/mL, particularly in PWH with other risk factors.

**Figure 1.**
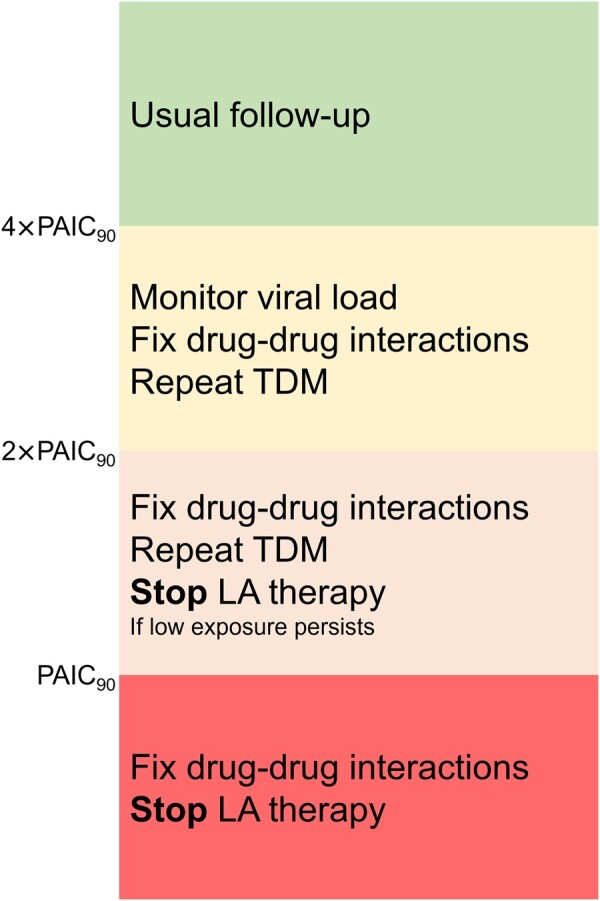
Flowchart for the guidance on the interpretation of long-acting (LA) cabotegravir and rilpivirine concentrations. Abbreviations: PAIC_90_, 90% inhibition of viral replication; TDM, therapeutic drug monitoring.

Based on these observations, we suggest the following guidance for interpretating LA cabotegravir-rilpivirine data and related monitoring, depending on cabotegravir and rilpivirine measurements (Figure 1).

1. Cabotegravir and/or rilpivirine C_trough_ measurements below PAIC_90_ (ie, 166 and 12 ng/mL): Such low drug exposures put persons at risk of VF. As usual with substantially reduced drug concentrations, drug-drug interactions with potent inducers of drug metabolizing enzymes (UGT1A1 for cabotegravir and CYP3A4 for rilpivirine) must first be excluded. If the coadministration of inducers is excluded, we then recommend stopping LA therapy if there is ≥1 associated baseline risk factor (ie, HIV-1 subtype A6/A1, BMI ≥30, rilpivirine resistance mutations at baseline, low C_trough_ 4 weeks after the initial loading dose) .
2. Cabotegravir and/or rilpivirine C_trough_ measurements below 2 × PAIC_90_ (ie, 332 and 24 ng/mL) but above PAIC_90_: Concentrations above the PAIC_90_ thresholds are in theory less threatening as these concentrations can still inhibit 90% of the viral replication based on in vitro measurements. Given the large interindividual but also intraindividual variability observed with intramuscular injections [8], we recommend repeating a C_trough_ measurement to confirm the suboptimal drug exposure. If ≥2 consecutive levels fall below the 2 × PAIC_90_ threshold in a person with HIV with ≥1 associated baseline risk factor, we then recommend discontinuing LA therapy. Such situations would indeed imply lower cabotegravir and/or rilpivirine concentrations for several weeks or months, thereby potentially increasing the risk of VF and resistance mutations. Low consecutive levels below 2 × PAIC_90_ were observed in our observational TDM study in only 1.5% of PWH on LA cabotegravir and in 6.4% of those on LA rilpivirine.
3. Cabotegravir and/or rilpivirine C_trough_ measurements below 4 × PAIC_90_ (ie, 664 and 50 ng/mL) but preferably above 3 × PAIC_90_ (ie, 498 ng/mL) for cabotegravir and Q1_Ctrough_ (ie, 32 ng/mL) for rilpivirine: As in the category above, TDM should be repeated. If the levels are confirmed, close viral load monitoring is recommended (ie, every 2 or 4 months as clinically appropriate). Based on our TDM data, rilpivirine levels are often <50 ng/mL. Therefore, this threshold may be too stringent, especially if cabotegravir levels are adequate and/or no other risk factors are present, in which case LA therapy should be pursued.

TDM represents an additional tool for the monitoring of PWH and may allow identification of individuals in whom LA therapy could potentially fail. However, TDM requires careful recording of dosing, actual sampling time, and information on covariates of interest (eg, BMI or virus subtype).

The rate of VF in our observational study, which is slightly higher than that reported by Orkin et al (ie, 1.4%) [1], may be partly due to enrollment bias. Because our study allowed physicians to have real-time information on cabotegravir-rilpivirine concentrations, PWH with “complex” situations may have been overrepresented. In our study, decisions to preemptively discontinue the LA therapy were made in 3 PWH based on repeated low levels of cabotegravir and/or rilpivirine (ie, below 2 × PAIC_90_ and even below PAIC_90_). Although the relationship between low drug concentrations and VF remains unclear, it can be argued that discontinuation decisions based on TDM may have prevented VF with the development of resistance to integrase or nonnucleoside reverse-transcriptase inhibitor classes.

The studies listed in Table 1 did not provide detailed information on repeated drug measurements. Therefore, the intraindividual variability could not be compared with that observed in our study. In addition, the sensitivity and specificity of the different C_trough_ thresholds in predicting VF could not be determined in our study owing to the small number of VF cases. Finally, the findings suggest that VF tends to occur several months after the initiation of LA treatment (Table 1). Although cohorts seem to favor drug level measurements during the first weeks of LA treatment, VF cases indicate that this timeline could not be adapted to detect suboptimal drug exposure in the medium long term and that longer monitoring should be performed.

In summary, available real-world data support the use of TDM for LA cabotegravir-rilpivirine therapy as it could prevent VF. TDM is particularly advised in obese or morbidly obese PWH or any other special populations underrepresented in clinical trials for which limited data are available on the exposure and response to LA cabotegravir-rilpivirine treatment.

## Supplementary Material

ofae023_Supplementary_Data

## Supplementary Data

Supplementary materials are available at *Open Forum Infectious Diseases* online. Consisting of data provided by the authors to benefit the reader, the posted materials are not copyedited and are the sole responsibility of the authors, so questions or comments should be addressed to the corresponding author.
