# Supplementary material for: Guidance for the Interpretation of Long-Acting Cabotegravir and Rilpivirine Concentrations Based on Real-World Therapeutic Drug Monitoring Data and Documented Failures
Source: Open Forum Infect Dis. 2024 Jan 16;11(2):ofae023. doi: 10.1093/ofid/ofae023 (PMC10878054; doi:10.1093/ofid/ofae023)
Supplement: ofae023_Supplementary_Data [file ofae023_supplementary_data.docx]

**
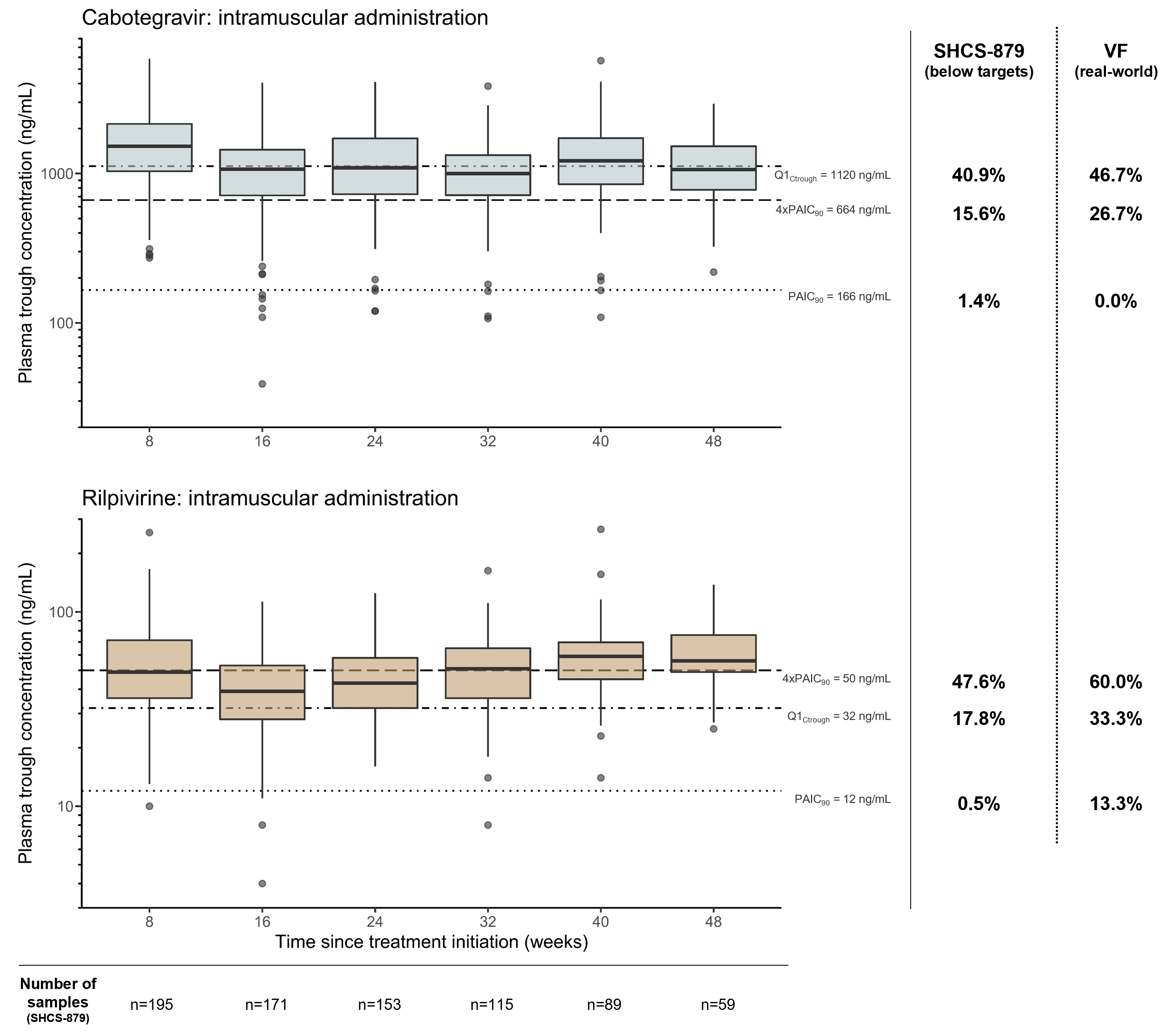
Supplementary Material**

Figure S1: Trough plasma concentrations of cabotegravir and rilpivirine observed in participants of the SHCS over the first 48 weeks of treatment. The boxplots depict the median and interquartile range, while whiskers extend from the lowest to the highest values comprised within 1.5 times the interquartile range. Outliers are represented by black dots. The horizontal lines correspond to the PAIC_90_ (dotted line), 4xPAIC_90_ (dashed line), and Q1_Ctrough_ (dot-dashed line) thresholds [2-7]. The percentage of samples below each threshold is shown on the right, while the total number of samples at each time point is shown at the bottom. The percentage of virologic failure at each threshold is also shown on the right.

Table S1: Proportion of PWH with measured cabotegravir and/or rilpivirine concentrations below the various thresholds

| Cabotegravir i.m. | PWH with no measurement | PWH with 1 measurement | PWH with ≥ 2 measurements |
| --- | --- | --- | --- |
| < 1120 ng/mL (Q1_Ctrough_)  < 664 ng/mL (4xPAIC_90_)  < 332 ng/mL (2xPAIC_90_)  ≤ 166 ng/mL (PAIC_90_) | 86 (33%)  162 (62%)  235 (89%)  251 (95%) | 67 (25%)  64 (24%)  21 (8%)  12 (5%) | 111 (42%)  38 (14%)  8 (3%)  1 (<1%) |
|  | | | |
| Rilpivirine i.m. | **PWH with no measurement** | **PWH with 1 measurement** | **PWH with ≥ 2 measurements** |
| < 50 ng/mL (4xPAIC_90_)  < 32 ng/mL (Q1_Ctrough_)  < 24 ng/mL (2xPAIC_90_)  ≤ 12 ng/mL (PAIC_90_) | 70 (27%)  166 (63%)  220 (83%)  259 (98%) | 67 (25%)  54 (20%)  28 (11%)  4 (2%) | 127 (48%)  44 (17%)  16 (6%)  1 (<1%) |

PWH: people with HIV; i.m.: intramuscular; PAIC_90_: protein-adjusted concentrations for 90% inhibition of viral replication; Q1C_trough_: upper limit of the first C_trough_ quartile derived from Phase III trials.


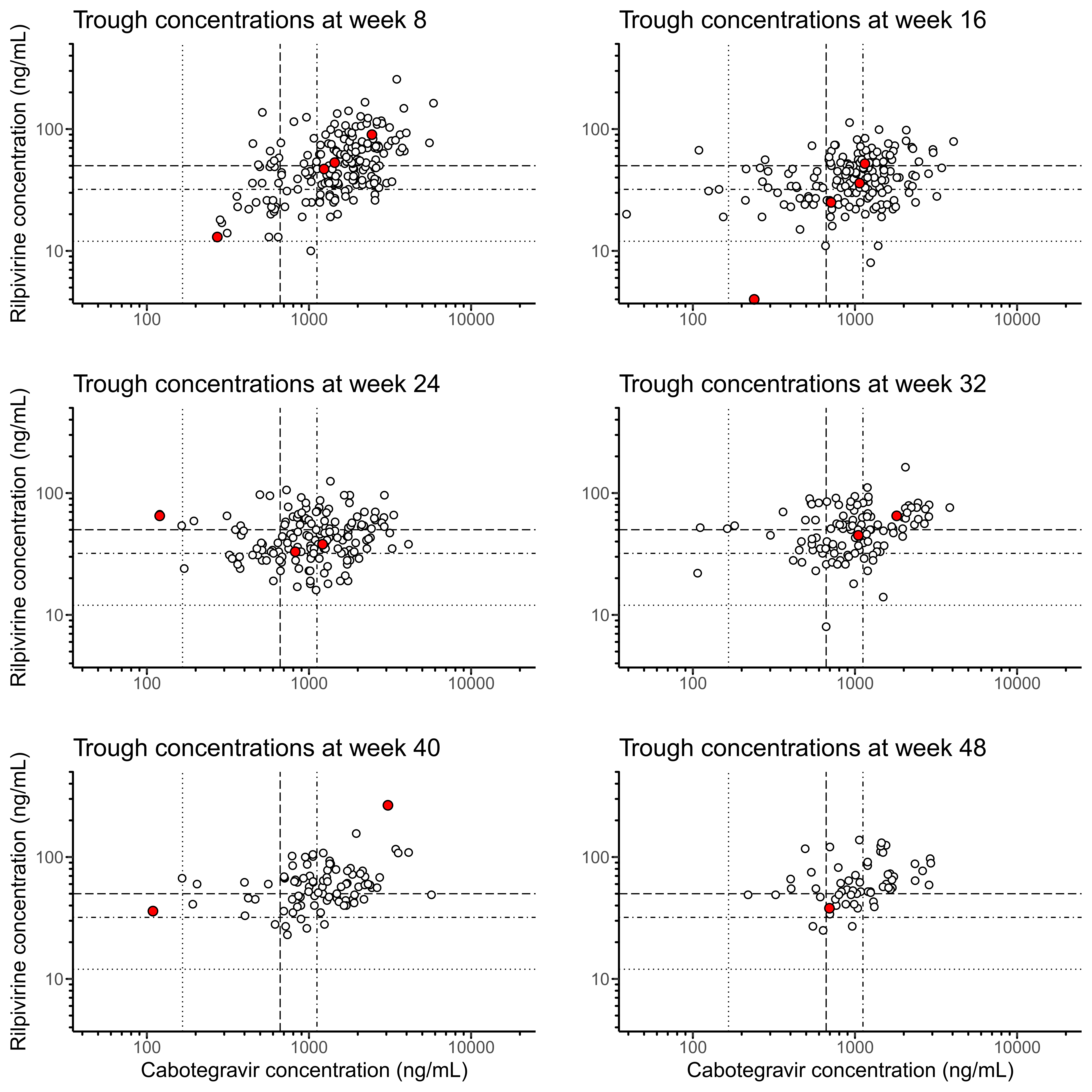


Figure S2: Cabotegravir and rilpivirine trough concentrations at week 8 (i.e., 4 weeks after the loading dose), week 16, week 24, week 32, week 40, and week 48. Red dots highlight the drug levels of SHCS PWH with virologic failure. The horizontal lines correspond to the PAIC_90_ (dotted line), 4xPAIC_90_ (dashed line), and Q1_Ctrough_ (dot-dashed line) thresholds [2-7].
